# Physical activity, falls, and dementia risk in adults aged ≥ 60: evidence from three cohorts

**DOI:** 10.1186/s12966-026-01926-9

**Published:** 2026-05-09

**Authors:** Juxiang Yang, Minheng Zhang, Miaomiao Hou, Hongwei Liu, Haixia Fan

**Affiliations:** 1 Southwest University, Chongqing, China; 2 Department of Gerontology, The First People’s Hospital of Jinzhong, Shanxi Medical University, Jinzhong, Shanxi Province China; 3 Department of Neurology, Tongji Shanxi Hospital, Shanxi Bethune Hospital, Shanxi Academy of Medical Sciences, Third Hospital of Shanxi Medical University, Tongji Shanxi Hospital, Taiyuan, Shanxi Province China; 4 Department of Neurology, Xuanwu Hospital of Capital Medical University, #45 Changchun Street, Xicheng District, Beijing, 100053 China; 5 Department of Sleep center, First Hospital of Shanxi Medical University, No. 85 Jiefang South Road, Taiyuan, Shanxi Province 030001 China

**Keywords:** Fall, Physical activity, Dementia, Old adults

## Abstract

**Background:**

Falls and physical inactivity are both linked to increased dementia risk, but their joint impact has not been well studied. It remains unclear whether physical activity can mitigate the elevated dementia risk after a fall and whether it also lowers the likelihood of future falls.

**Methods:**

We used data from 44,488 adults aged ≥ 60 years in three cohorts. Falls and physical activity were self-reported. Incident dementia was tracked during follow-up. Cox proportional hazards models estimated the associations of falls with dementia and physical activity with dementia and falls, stratified by fall history.

**Results:**

Over a median follow-up of 5.9–7.9 years, 3,492 dementia cases were identified. Falls were associated with a 70% higher risk of dementia (pooled HR = 1.70, 95% CI: 1.57–1.84). In the no-fall group, compared with inactivity, low, moderate, and high physical activity were progressively associated with lower risks of dementia (HRs = 0.63, 0.53, 0.43) and falls (HRs = 0.77, 0.68, 0.58). These protective effects were consistent among fallers, with similar dose–response gradients.

**Conclusions:**

Falls substantially increased dementia risk. Higher levels of physical activity were linked to lower risks of both dementia and falls, regardless of fall history.

**Graphical abstract:**

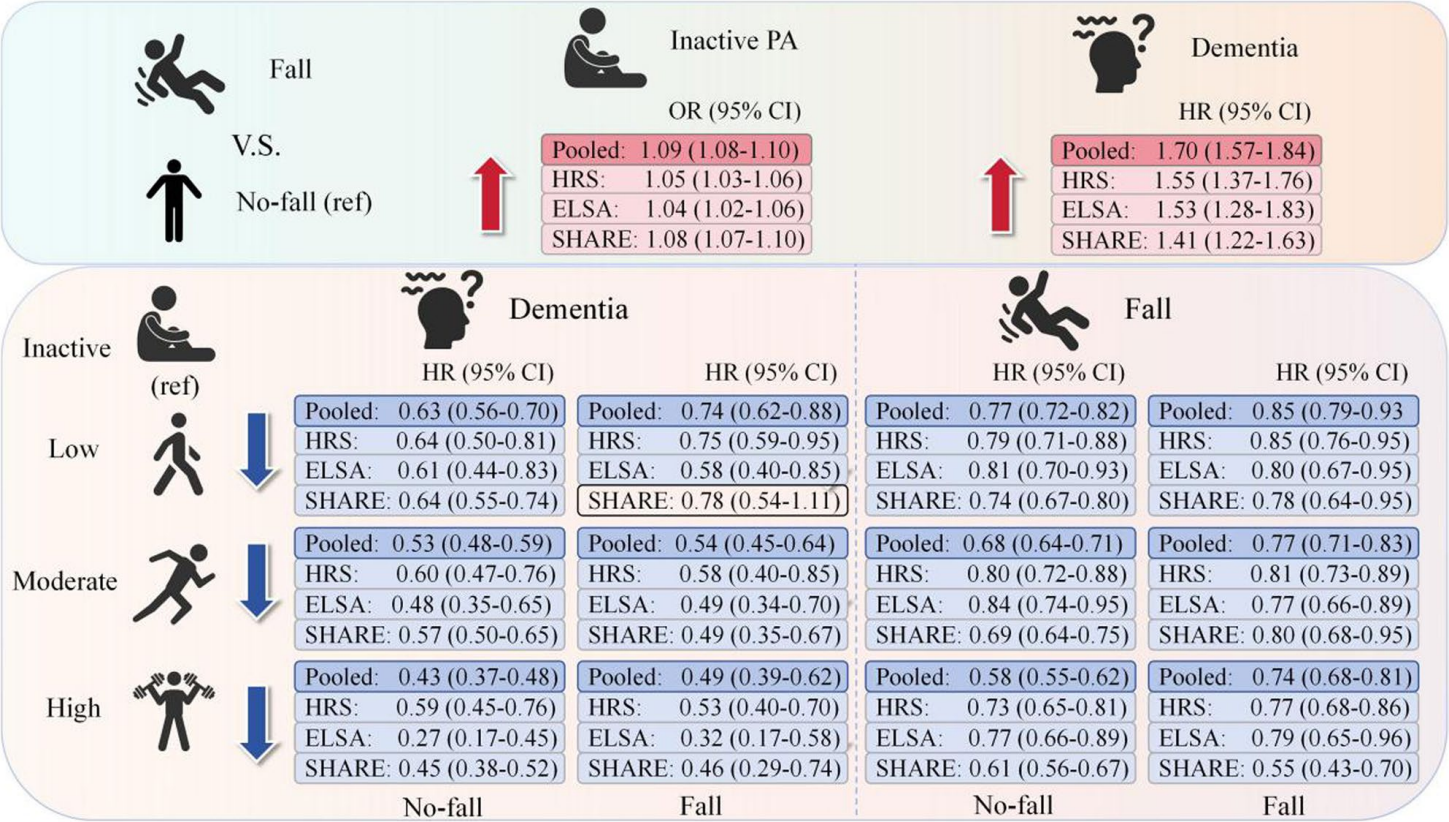

**Supplementary Information:**

The online version contains supplementary material available at 10.1186/s12966-026-01926-9.

## Question

Can physical activity lower the risk of both future falls and dementia, even after a previous fall?

## Key finding

Higher physical activity levels are associated with significantly lower risks of subsequent falls and incident dementia, regardless of fall history.

## Research significance

Encouraging physical activity, both routinely and after a fall, may help prevent falls and reduce dementia risk in older adults.

Physical Activity, Falls, and Dementia Risk in Adults Aged ≥ 60. HRS, the Health and Retirement Study; ELSA, English Longitudinal Study of Ageing; SHARE, Survey of Health, Ageing and Retirement in Europe; HR, hazard ratio; CI, confidence interva.

## Introduction

With the rapid acceleration of global population aging, dementia has emerged as a major public health concern [1, 2]. An estimated 57 million people worldwide are currently living with dementia, and more than 10 million new cases are diagnosed each year, placing a substantial burden on families, healthcare systems, and societies at large [3, 4]. Identifying modifiable risk factors for dementia is therefore a priority for prevention and health policy [5]. 

Falls are among the most common and serious health problems in older adults [6], affecting around one in three individuals aged 65 years and older, and up to half of those over 80 [7, 8]. Beyond injuries and disability, accumulating evidence indicates that falls may accelerate cognitive decline and significantly increase the risk of dementia [9, 10]. Given the high prevalence of falls and their profound health consequences, strategies to prevent both falls and fall-related cognitive decline are of great importance.

Physical activity is well established as a key protective factor against both dementia and falls [11–13]. A wide range of studies, using both self-reported and device-measured assessments, consistently show that higher levels of physical activity are associated with lower dementia incidence [14–17]. Importantly, physical activity also plays a central role in maintaining balance, muscle strength, and functional independence, thereby reducing the likelihood of falls [18, 19]. Despite these benefits, older adults who experience falls often reduce their activity levels due to fear of falling [20, 21], which may further exacerbate cognitive vulnerability.

Taken together, these findings raise two important but underexplored questions. First, how strongly do falls themselves contribute to subsequent dementia risk? Second, can physical activity mitigate these risks not only among those with a history of falls but also in older adults without prior falls? Addressing these questions has critical implications for designing preventive strategies that simultaneously target fall prevention and cognitive health promotion.

To answer these questions, we analyzed data from three large longitudinal cohorts (detailed in Methods). By examining the joint relationships of falls, physical activity, and dementia, we aimed to provide robust evidence on whether physical activity offers protective effects against both falls and dementia regardless of fall history.

## Methods

### Study design and participants

We selected the Health and Retirement Study (HRS), the English Longitudinal Study of Ageing (ELSA), and the Survey of Health, Ageing and Retirement in Europe (SHARE), because they are well-established, nationally representative aging cohorts conducted in the United States, the United Kingdom, and multiple European countries, respectively. These cohorts capture diverse socioeconomic, healthcare, and cultural contexts, providing an important opportunity to assess the generalizability of findings across different populations. In addition, they are part of a family of harmonized international aging studies with comparable study designs, sampling strategies, and survey instruments, and the availability of harmonized data from the Gateway to Global Aging Data enables consistent definitions of key variables across cohorts.

All three cohorts provide longitudinal data with repeated assessments of falls, physical activity, and cognitive function, allowing for the investigation of temporal relationships with incident dementia. In line with previous cross-national studies [22–24], we selected survey waves that were as closely aligned in calendar time as possible across cohorts to enhance comparability and minimize potential bias due to period effects.

All three cohorts provide longitudinal data with repeated assessments of falls, physical activity, and cognitive function, allowing for the investigation of temporal relationships with incident dementia. The baseline waves included HRS Wave 11 (2012), ELSA Wave 5 (2010–2011), and SHARE Wave 4 (2011). In SHARE Wave 4, participants from 16 European countries were surveyed, including Austria, Belgium, Switzerland, Germany, Denmark, Spain, France, Italy, the Netherlands, Sweden, the Czech Republic (Czechia), Ireland, Estonia, Hungary, Portugal, and Slovenia. Participants were followed through subsequent survey waves until the latest available waves: HRS Wave 15 (2020), ELSA Wave 9 (2018–2019), and SHARE Wave 8 (2020).

**Fig. 1:**
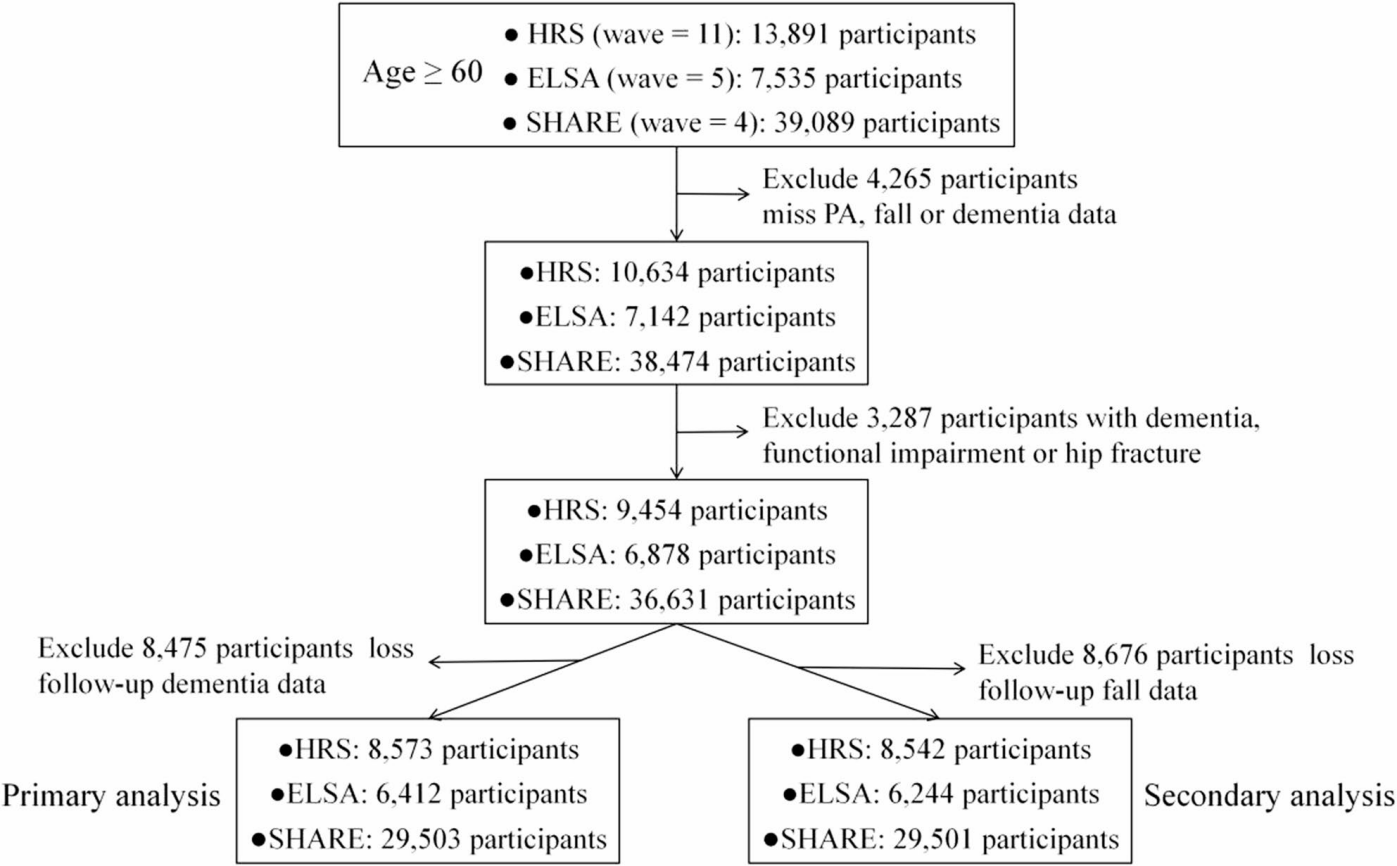
Selection process of the study population

Individuals aged ≥ 60 years at baseline were eligible. We excluded participants with missing data on physical activity, falls, or dementia, as well as those with baseline dementia, functional impairment, or hip fracture. For the primary analysis, we additionally excluded participants without follow-up dementia data; for the secondary analysis, those without follow-up fall data were excluded. The overall selection process is shown in Fig. 1. A total of 44,488 participants were included in the primary analysis (HRS: 8,573; ELSA: 6,412; SHARE: 29,503). The median follow-up time was 6.2 years overall, 7.6 years in HRS, 7.9 years in ELSA, and 5.9 years in SHARE. During follow-up, 3,492 incident dementia cases were documented, corresponding to an incidence rate of 39.4 per 1,000 person-years (HRS: 59.9, ELSA: 38.9, SHARE: 33.6).

The HRS, ELSA, and SHARE studies were approved by the University of Michigan [25], the London Multi-Centre Research [26], and the University of Mannheim [27], respectively. Since we utilized publicly available datasets, no additional ethical approval was required.

### Fall

Self-reported data were used to assess falls, a method widely applied in previous research. In HRS and ELSA, participants aged ≥ 60 years were asked: “Have you fallen down in the past two years (for any reason)?” or, if they had participated in the prior wave, “Have you fallen down since your last interview?” (yes/no) [28, 29]. In SHARE, falls were identified by asking: “For the past six months at least, have you been bothered by any of the health conditions on this card?” with “falling down” listed among five conditions on a showcard [30]. Across cohorts, a “yes” response defined a fall, and “no” defined no-fall.

### Physical Activity (PA)

Each cohort provided participants with specific examples to help them differentiate between levels of physical activity (PA) intensity, building upon the International Physical Activity Questionnaire (IPAQ) [31]. PA was assessed by asking participants how often they engaged in vigorous, moderate, and mild activities. Before answering, participants were given examples to clarify the intensity categories. Examples of moderate-intensity activities included gardening, cleaning the car, walking at a moderate pace, dancing, and floor or stretching exercises, whereas vigorous-intensity activities included running or jogging, swimming, cycling, aerobics or gym workouts, tennis, and digging with a spade. For each intensity level, participants chose from four response options: rarely or never, one to three times per month, once a week, or multiple times per week [26]. 

To ensure comparability across cohorts, PA levels were harmonized according to established criteria from previous research [32, 33]. Participants were classified into four categories: 1). Inactive: No moderate or vigorous physical activity on a weekly basis. 2). Low: Mild physical activity at least once per week, or moderate physical exercise once a week or less, with no vigorous physical activity. 3). Moderate: Engagement in physical work, or moderate physical activity more than once per week combined with vigorous physical activity once a week or less. 4). High: Participation in heavy manual work or vigorous physical activity more than once per week.

### Dementia

Dementia was defined using two criteria: 1). Physician-diagnosed dementia: Participants were asked whether a doctor had ever diagnosed them with Alzheimer’s disease, dementia, or senile dementia [34]. 2). Combined cognitive and functional impairment: memory, numeracy, and orientation. Memory was assessed using immediate and delayed word recall tests modified from Rey’s Auditory Verbal Learning Test (range 0–20). Numeracy was measured by the Serial Sevens subtraction test (range 0–5), and orientation was evaluated by naming the month, date, day of the week, and year (range 0–4). A global cognitive score (range 0–29) was created by summing the three domains [35], with cognitive impairment defined as a score ≥ 1.5 standard deviations below the mean, adjusted for age and educational level [36]. Functional impairment was defined as difficulty performing at least one basic activity of daily living (bathing, eating, dressing, transferring in and out of bed, or walking across a room) [37]. Participants meeting either criterion were classified as having dementia, in accordance with prior validations across the included cohorts [34–37]. 

### Covariates

Covariates were selected based on previous literature. Demographic variables included age, sex (male/female), marital status (married or partnered vs. single), and education level (below high school, high school, or college and above) [38]. Lifestyle factors included smoking status (never, former, or current) and alcohol consumption (yes/no) [39]. Chronic disease history was based on self-report of physician-diagnosed conditions and included hypertension, diabetes, cancer, chronic lung disease, heart disease, stroke, arthritis, and osteoporosis [40]. To ensure cross-cohort consistency, only conditions assessed in all cohorts were included. Depressive symptoms were assessed using cohort-specific validated scales: HRS and ELSA used the 8-item Center for Epidemiologic Studies Depression Scale (CES-D8) with a cut-off score of ≥ 3, while SHARE applied the 12-item EURO-D scale with a cut-off score of ≥ 4 [22]. 

Following previous cross-national studies that addressed missing covariate data using multiple imputation methods [38], we performed multiple imputation by chained equations (MICE) using the mice package in R (version 4.3.2). The imputation model incorporated all relevant covariates and the outcome and exposure variables. Twenty imputed datasets were created using predictive mean matching, and results were combined using Rubin’s rules. Participants with missing data on exposure or outcome were excluded prior to imputation.

### Data harmonization

To ensure comparability across cohorts, we used the harmonized datasets provided by the Gateway to Global Aging Data project, which contain standardized variable definitions and detailed documentation for HRS, ELSA, and SHARE. Using these harmonized variables minimized cross-cohort measurement discrepancies and allowed us to apply consistent definitions for key variables.

### Statistical analysis

Continuous variables with normal distribution were summarized as means ± standard deviations and compared using Student’s t-tests. Categorical variables were presented as frequencies (percentages) and compared using chi-square or Fisher’s exact tests, as appropriate. A *P* value < 0.05 was considered statistically significant.

All three cohorts recorded the year and month of each interview. For each participant, time to event was calculated from the baseline interview date to either the first report of dementia or censoring (defined as the date of last available follow-up without dementia diagnosis). Cox proportional hazards models were used to estimate hazard ratios (HRs) and 95% confidence intervals (CIs) for the associations of falls and physical activity with incident dementia. Models were stratified by fall history to examine whether the association between physical activity and dementia differed between participants with and without prior falls. The proportional hazards assumption was tested using Schoenfeld residuals, and no violations were detected for the main exposures.

We conducted separate models for pooled data and for each cohort (HRS, ELSA, and SHARE). Three progressively adjusted models were fitted: Model 1 was not adjusted. Model 2 was adjusted for gender, age, education level and marital status. Model 3 was adjusted for Model 2 plus smoking status, drinking, osteoporosis, hypertension, diabetes, cancer, chronic lung disease, heart disease, stroke and arthritis. In addition, following prior studies [41], we conducted pooled analyses by combining individual-level data from all cohorts. This approach allowed us to maximize statistical power, ensure consistent covariate adjustment, and evaluate overall associations across diverse populations. Cluster-robust standard errors were used to account for within-study correlation. To evaluate the robustness of findings, we performed covariate-adjusted subgroup analyses using the pooled dataset, assessing whether the association between physical activity and dementia differed across population subgroups.

All statistical analyses were two-sided and conducted using R software (version 4.3.2; www.r-project.org).

### Equity, Diversity, and Inclusion (EDI) statement

In alignment with the commitment to equity, diversity, and inclusion (EDI) [42], this study incorporates data from multiple countries, including the United States, the United Kingdom, and European regions, to enhance diversity in socioeconomic, cultural, and healthcare contexts. By leveraging harmonized international aging cohorts, our analysis aims to improve the generalizability of findings and avoid biases associated with single-country studies. This cross-national approach also allows for a more inclusive understanding of how falls, physical activity, and dementia risk may vary across different population settings. However, we acknowledge that the three cohorts predominantly include participants of White ethnicity and from high-income countries (USA, UK, and Europe). This limits the generalizability of our findings to more diverse racial/ethnic groups and low- or middle-income settings. Future research should prioritize more inclusive samples.

## Results

### Baseline characteristics of the study population

This study included 44,488 participants from three large population-based cohorts: HRS (*n* = 8,573), ELSA (*n* = 6,412), and SHARE (*n* = 29,503). Participants were categorized into fall and non-fall groups based on their baseline fall history.

Baseline characteristics stratified by fall status are summarized in Table 1. Across all cohorts, fallers were significantly older than non-fallers (e.g., SHARE: 73.98 ± 8.40 vs. 69.94 ± 7.34 years; *p* < 0.001) and less likely to be male (e.g., SHARE: 23.9% vs. 44.7%; *p* < 0.001). Fallers were also less likely to be married or partnered and, in ELSA and SHARE, more likely to have education below the high-school level (*p* < 0.001 for both). Lifestyle characteristics showed mixed associations with fall status, but physical inactivity was consistently more prevalent among fallers across all cohorts (*p* < 0.001).

**Table 1:** Baseline characteristics of 44,488 participants from three cohort studies by fall group

Variables	HRS (8573)			ELSA (6412)			SHARE (29503)		
	No-Fall (*n* = 5713)	Fall (*n* = 2860)	*P*	No-Fall (*n* = 4618)	Fall (*n* = 1794)	*P*	No-Fall (*n* = 28004)	Fall (*n* = 1499)	*P*
Age, Mean ± SD	74.56 ± 6.54	75.89 ± 7.13	<0.001	69.64 ± 7.33	71.61 ± 8.19	<0.001	69.94 ± 7.34	73.98 ± 8.40	<0.001
Gender (Male), n (%)	2446 (42.8)	1088 (38)	<0.001	2187 (47.4)	718 (40.0)	<0.001	12,513 (44.7)	359 (23.9)	<0.001
Marital status, n (%)			<0.001			<0.001			<0.001
Married or partnered	3313 (58.0)	1542 (53.9)		3139 (68)	1056 (58.9)		19,114 (68.3)	785 (52.4)	
Other marital status	2400 (42.0)	1318 (46.1)		1479 (32)	738 (41.1)		8890 (31.7)	714 (47.6)	
Education, n (%)			0.499			<0.001			<0.001
Below high school	1167 (20.4)	578 (20.2)		1597 (34.6)	707 (39.4)		12,827 (45.8)	875 (58.4)	
High school	3305 (57.9)	1689 (59.1)		2269 (49.1)	838 (46.7)		9928 (35.5)	410 (27.4)	
College or above	1241 (21.7)	593 (20.7)		752 (16.3)	249 (13.9)		5249 (18.7)	214 (14.3)	
Drinking, n (%)	2892 (50.6)	1384 (48.4)	0.052	2962 (64.1)	1044 (58.2)	<0.001	12,874 (46)	483 (32.2)	<0.001
Smoking, n (%)			<0.001			0.003			<0.001
Non-smoker	2519 (44.1)	1272 (44.5)		1735 (37.6)	599 (33.4)		15,806 (56.4)	983 (65.6)	
Former smoker	2670 (46.7)	1395 (48.8)		2418 (52.4)	981 (54.7)		8210 (29.3)	349 (23.3)	
Current smoker	524 (9.2)	193 (6.7)		465 (10.1)	214 (11.9)		3988 (14.2)	167 (11.1)	
Physical activity, n (%)			<0.001			<0.001			<0.001
Inactive	1063 (18.6)	779 (27.2)		686 (14.9)	461 (25.7)		3033 (10.8)	428 (28.6)	
Low	1577 (27.6)	712 (24.9)		996 (21.6)	358 (20.0)		5080 (18.1)	282 (18.8)	
Moderate	1645 (28.8)	798 (27.9)		2070 (44.8)	713 (39.7)		11,321 (40.4)	553 (36.9)	
High	1428 (25.0)	571 (20.0)		866 (18.8)	262 (14.6)		8570 (30.6)	236 (15.7)	
Osteoporosis, n (%)	879 (15.4)	576 (20.1)	<0.001	321 (7)	195 (10.9)	<0.001	6606 (23.6)	698 (46.6)	<0.001
Hypertension, n (%)	3794 (66.4)	1983 (69.3)	<0.001	2044 (44.3)	930 (51.8)	<0.001	13,532 (48.3)	925 (61.7)	<0.001
Diabetes, n (%)	1353 (23.7)	805 (28.1)	<0.001	505 (10.9)	227 (12.7)	0.052	4011 (14.3)	351 (23.4)	<0.001
Cancer, n (%)	1058 (18.5)	589 (20.6)	0.021	518 (11.2)	184 (10.3)	0.269	2284 (8.2)	159 (10.6)	<0.001
Lung disease, n (%)	544 (9.5)	354 (12.4)	<0.001	259 (5.6)	160 (8.9)	<0.001	2275 (8.1)	219 (14.6)	<0.001
Heart disease, n (%)	1506 (26.4)	1043 (36.5)	<0.001	833 (18)	441 (24.6)	<0.001	5140 (18.4)	488 (32.6)	<0.001
Stroke, n (%)	471 (8.2)	369 (12.9)	<0.001	166 (3.6)	130 (7.2)	<0.001	1459 (5.2)	199 (13.3)	<0.001
Arthritis, n (%)	3669 (64.2)	2189 (76.5)	<0.001	1748 (37.9)	889 (49.6)	<0.001	8597 (30.7)	844 (56.3)	<0.001
Depressive symptom	851 (14.9)	667 (23.3)	<0.001	805 (17.4)	551 (30.7)	<0.001	7361 (26.3)	842 (56.2)	<0.001

The differences between categorical variables were assessed using χ2 tests, and the differences between continuous variables were assessed using t tests

*HRS* Health and Retirement Study, *ELSA* English Longitudinal Study of Ageing, *SHARE* Survey of Health, Ageing and Retirement in Europe.

### Association of falls with physical activity and dementia risk

To investigate the relationship between falls, physical activity, and dementia risk, we compared physical activity levels between fallers and non-fallers and applied multivariable logistic regression to estimate the odds of physical inactivity and Cox regression to estimate the risk of incident dementia, adjusting for sociodemographic, lifestyle, and health-related factors.

**Fig. 2:**
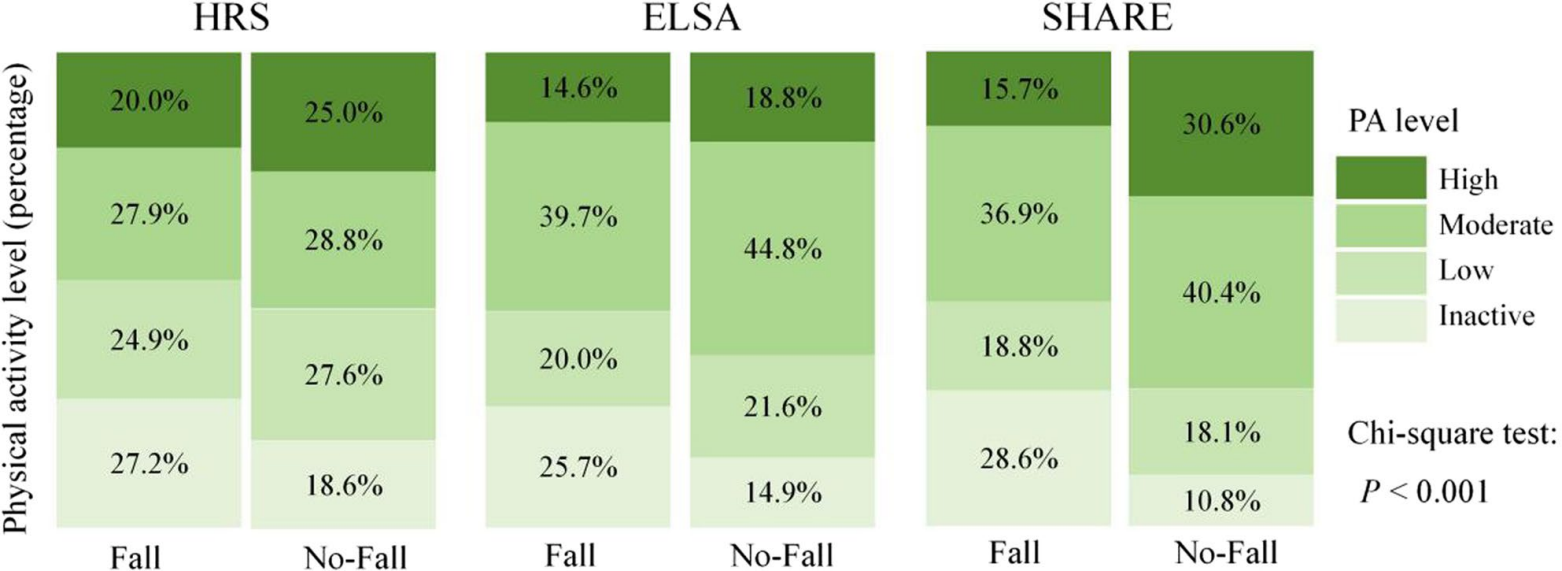
Distribution of Physical Activity Levels by Cohort and Fall Status

The distribution of physical activity differed significantly between fallers and non-fallers (all *P* < 0.001; e.g., 28.6% vs. 10.8%), with a higher proportion of fallers classified as physically inactive (Fig. 2). In the model 3 (Table 2), falls were associated with higher odds of physical inactivity (OR = 1.09, 95% CI: 1.08–1.10), with similar results observed across HRS (OR = 1.05), ELSA (OR = 1.04), and SHARE (OR = 1.08). Falls were also significantly associated with an increased risk of incident dementia (pooled HR = 1.09, 95% CI: 1.08–1.10), with consistent cohort-specific results (HRS: HR = 1.05; ELSA: HR = 1.04; SHARE: HR = 1.08; all *P* < 0.001).

In summary, older adults with a history of falls were more likely to be physically inactive and had a higher risk of incident dementia compared with those without falls.

**Table 2:** Association of Falls with Physical Inactivity and Dementia Risk

Fall	Physical Inactivity : OR (95%CI)			Dementia risk : HR(95%CI)		
	Model 1	Model 2	Model 3	Model 1	Model 2	Model 3
Pooled						
No	1.00 (reference)	1.00 (reference)	1.00 (reference)	1.00 (reference)	1.00 (reference)	1.00 (reference)
Yes	1.16 (1.15-1.17)**	1.12 (1.11-1.13)**	1.09 (1.08-1.10)**	1.16 (1.15-1.17)**	1.12 (1.11-1.13)**	1.09 (1.08-1.10)**
HRS						
No	1.00 (reference)	1.00 (reference)	1.00 (reference)	1.00 (reference)	1.00 (reference)	1.00 (reference)
Yes	1.09 (1.07-1.11)**	1.07 (1.06-1.09)**	1.05 (1.03-1.06)**	1.09 (1.07-1.11)**	1.07 (1.06-1.09)**	1.05 (1.03-1.06)**
ELSA						
No	1.00 (reference)	1.00 (reference)	1.00 (reference)	1.00 (reference)	1.00 (reference)	1.00 (reference)
Yes	1.11 (1.09-1.14)**	1.08 (1.06-1.10)**	1.04 (1.02-1.06)**	1.11 (1.09-1.14)**	1.08 (1.06-1.10)**	1.04 (1.02-1.06)**
SHARE						
No	1.00 (reference)	1.00 (reference)	1.00 (reference)	1.00 (reference)	1.00 (reference)	1.00 (reference)
Yes	1.19 (1.17-1.21)**	1.14 (1.12-1.16)**	1.08 (1.07-1.10)**	1.19 (1.17-1.21)**	1.14 (1.12-1.16)**	1.08 (1.07-1.10)**

Model 1 was not adjusted. Model 2 was adjusted for gender, age, education level and marital status. Model 3 was adjusted for Model 2 plus smoking status, drinking, chronic disease and depressive symptom

*HRS* Health and Retirement Study, *ELSA* English Longitudinal Study of Ageing, *SHARE* Survey of Health, Ageing and Retirement in Europe

*P < 0.05,**P < 0.001

### Associations of physical activity with incident falls and dementia, stratified by baseline fall status

To examine the associations of physical activity with dementia and fall risk, we conducted analyses stratified by baseline fall status using multivariable Cox proportional hazards models adjusted for sociodemographic, lifestyle, and health-related factors.

As shown in Table 3, higher physical activity levels were consistently associated with a lower risk of dementia among participants with and without a history of falls (all P-trend < 0.001). In the pooled analysis of participants without falls, compared with those who were inactive, low (HR = 0.63; 95% CI: 0.56–0.70), moderate (HR = 0.53; 95% CI: 0.48–0.59), and high (HR = 0.43; 95% CI: 0.37–0.48) activity were associated with progressively lower dementia risk. A similar graded association was observed among fallers, although in the SHARE cohort the association for low activity was not statistically significant (HR = 0.78; 95% CI: 0.54–1.11).

Table 4 shows that physical activity was also inversely associated with incident falls. Among non-fallers, higher activity levels were associated with progressively lower fall risk (pooled HR = 0.77 for low, 0.68 for moderate, 0.58 for high; all P-trend < 0.001). Among fallers, higher physical activity remained protective, with significant trends in HRS, SHARE, and the pooled analysis (all P-trend < 0.001), and a weaker but significant trend in ELSA (P-trend = 0.015).

Overall, higher physical activity was associated with both reduced risk of dementia and lower risk of subsequent falls, regardless of baseline fall status.

**Table 3:** Association of physical activity with risks of dementia (group by fall)

PA	HRS	ELSA	SHARE	Pooled
	HR (95%CI)			
No-fall				
Inactive	1.00 (reference)	1.00 (reference)	1.00 (reference)	1.00 (reference)
Low	0.64 (0.50-0.81)**	0.61 (0.44-0.83)**	0.64 (0.55-0.74)**	0.63 (0.56-0.70)**
Moderate	0.60 (0.47-0.76)**	0.48 (0.35-0.65)**	0.57 (0.50-0.65)**	0.53 (0.48-0.59)**
High	0.59 (0.45-0.76)**	0.27 (0.17-0.45)**	0.45 (0.38-0.52)**	0.43 (0.37-0.48)**
	P-trend < 0.001	P-trend < 0.001	P-trend < 0.001	P-trend < 0.001
Fall				
Inactive	1.00 (reference)	1.00 (reference)	1.00 (reference)	1.00 (reference)
Low	0.75 (0.59-0.95)*	0.58 (0.40-0.85)*	0.78 (0.54-1.11)	0.74 (0.62-0.88)*
Moderate	0.58 (0.45-0.74)**	0.49 (0.34-0.70)**	0.49 (0.35-0.67)**	0.54 (0.45-0.64)**
High	0.53 (0.40-0.70)**	0.32 (0.17-0.58)**	0.46 (0.29-0.74)**	0.49 (0.39-0.62)**
	P-trend < 0.001	P-trend < 0.001	P-trend < 0.001	P-trend < 0.001

Model was adjusted for gender, age, education level, marital status, smoking status, drinking, chronic disease and depressive symptom

*HRS* Health and Retirement Study, *ELSA* English Longitudinal Study of Ageing, *SHARE* Survey of Health, Ageing and Retirement in Europe

*P < 0.05,**P < 0.001

**Table 4:** Association of physical activity with risks of fall (group by fall)

PA	HRS	ELSA	SHARE	Pooled
	HR (95%CI)			
No-fall				
Inactive	1.00 (reference)	1.00 (reference)	1.00 (reference)	1.00 (reference)
Low	0.79 (0.71-0.88)**	0.81 (0.70-0.93)*	0.74 (0.67-0.80)**	0.77 (0.72-0.82)**
Moderate	0.80 (0.72-0.88)**	0.84 (0.74-0.95)*	0.69 (0.64-0.75)**	0.68 (0.64-0.71)**
High	0.73 (0.65-0.81)**	0.77 (0.66-0.89)**	0.61 (0.56-0.67)**	0.58 (0.55-0.62)**
	P-trend < 0.001	P-trend < 0.001	P-trend < 0.001	P-trend < 0.001
Fall				
Inactive	1.00 (reference)	1.00 (reference)	1.00 (reference)	1.00 (reference)
Low	0.85 (0.76-0.95)*	0.80 (0.67-0.95)*	0.78 (0.64-0.95)*	0.85 (0.79-0.93)**
Moderate	0.81 (0.73-0.89)**	0.77 (0.66-0.89)*	0.80 (0.68-0.95)*	0.77 (0.71-0.83)**
High	0.77 (0.68-0.86)**	0.79 (0.65-0.96)*	0.55 (0.43-0.70)**	0.74 (0.68-0.81)**
	P-trend < 0.001	P-trend =0.015	P-trend < 0.001	P-trend < 0.001

Model was adjusted for gender, age, education level, marital status, smoking status, drinking, chronic disease and depressive symptom

*HRS* Health and Retirement Study, *ELSA* English Longitudinal Study of Ageing, *SHARE* Survey of Health, Ageing and Retirement in Europe

*P < 0.05,**P < 0.001

### Sensitivity analyses

To assess the robustness of our findings, we performed several sensitivity analyses. First, we repeated the primary analyses using two alternative dementia definitions: physician-diagnosed dementia and a combined cognitive and functional impairment measure. The protective association of physical activity with incident dementia remained significant in both definitions (Supplementary Table 1). For example, among participants without a history of falls, high physical activity was associated with a 59% lower risk of dementia based on physician diagnosis (HR = 0.41, 95% CI: 0.34–0.46) and a 58% lower risk using the combined definition (HR = 0.42, 95% CI: 0.35–0.48).

Second, we excluded participants with a history of stroke and those who developed dementia within the first two years of follow-up to minimize reverse causation. The results were materially unchanged, with high physical activity still linked to nearly 45% lower dementia risk (HR = 0.55, 95% CI: 0.43–0.69) in the fall group (Supplementary Table 1).

Third, because mortality data were available in HRS and SHARE, we repeated the analyses considering the competing risk of all-cause death (i.e., death from any cause before dementia diagnosis, which may bias incidence estimates). We applied Fine-Gray models treating all-cause death as a competing event (Supplementary Table 2). The protective associations of moderate-to-high physical activity persisted. For instance, in the fall group, high physical activity was associated with a 36% lower dementia risk in HRS (HR = 0.64, 95% CI: 0.48–0.86) and a 45% lower risk in SHARE (HR = 0.55, 95% CI: 0.35–0.86).

Finally, subgroup analyses among participants with a history of falls showed that the inverse association between post-fall physical activity and dementia was broadly consistent across strata of sex, marital status, education, alcohol use, smoking, major chronic conditions, and depressive symptoms (Supplementary Table 3). These findings reinforce the robustness and generalizability of our results.

## Discussion

Falls and dementia have long been recognized as critical issues in the context of healthy aging and are becoming increasingly important with global population aging [43–45]. Physical activity is widely regarded as a preventive strategy for both falls and dementia [11], and has been shown to slow the decline in ADL independence and improve balance among cognitively unimpaired individuals [46]. However, it remains unclear whether physical activity is associated with the risk of dementia and future falls among older adults with and without a history of falls.

To address this gap, we utilized data from three large-scale longitudinal cohorts (HRS, ELSA, and SHARE) to examine the relationship between falls, physical activity levels and dementia risk in older adults. In this large multicohort analysis of more than 44,000 older adults, we found that falls were associated with a 70% higher risk of developing dementia. Importantly, higher levels of physical activity were associated with lower risks of both dementia and future falls, with consistent dose–response patterns observed in individuals with and without a history of falls. These associations persisted after adjustment for demographic and health-related factors, underscoring the potential of physical activity as a modifiable target for dementia prevention, even among those who have already experienced a fall.

Of note, physical activity levels exhibited marked variation across the three cohorts, with a higher proportion of participants engaging in high-level PA in SHARE relative to HRS and ELSA. These differences likely reflect the combined influence of socio-cultural and environmental determinants. For instance, active lifestyles in retirement are more culturally embedded in Southern Europe, where routine walking and gardening are common and were captured as moderate PA. Furthermore, the walkable urban design of many European cities may facilitate higher daily ambulation compared to the car-dependent suburban environments typical of the US (HRS) and the UK (ELSA) [47]. By contrast, the US cohort had higher burdens of obesity and comorbidity, which may restrict engagement in vigorous PA [48]. Notwithstanding these contextual differences, the consistent dose-response relationship between PA and reduced risks of dementia and falls across all three cohorts reinforces the universal relevance of promoting physical activity in ageing populations.

Physical activity may exert protective effects against dementia through multiple biological mechanisms. Regular exercise has been linked to increased regional cerebral blood flow, particularly in the hippocampus and gray matter—areas essential for memory and attention. Cellular mechanisms may involve the upregulation of neurobiological factors such as brain-derived neurotrophic factor (BDNF) and myokines, which promote hippocampal neuroplasticity [49]. BDNF, in particular, is known to support synaptic plasticity, neurogenesis, and cognitive function [50]. Additional benefits may derive from improved hemodynamics, including enhanced endothelial nitric oxide synthase (eNOS) phosphorylation, reduced oxidative stress, angiogenesis, and increased cerebral perfusion [51]. 

In addition to cognitive benefits, physical activity is a well-established intervention to prevent falls through multiple pathways. Regular physical activity may alleviate the psychological sequelae of falling—such as fear of falling, social withdrawal, and reduced activity—which themselves are associated with accelerated cognitive decline. It is important to note that previous studies have found that physical activity interventions may not significantly reduce fall risk or improve ADL in individuals with established dementia [52–54]. This underscores the importance of early intervention in the pre-dementia phase, particularly among older adults with a fall history. Identifying fallers and supporting them to maintain or increase their physical activity may be crucial for breaking the cycle between falls, inactivity, and dementia.

### Clinical and policy implications

These findings have important implications for clinical practice and public health policy. Older adults who have experienced a fall are more likely to be physically inactive and have a higher risk of developing dementia, highlighting the need to incorporate fall history into routine risk assessments for both physical and cognitive health. In clinical settings, encouraging and supporting safe physical activity after a fall should be prioritized, not only to reduce the likelihood of recurrent falls but also to mitigate dementia risk. To translate these findings into routine care, we propose several actionable strategies. First, fall history should be systematically documented in primary care as a simple screener (e.g., “Have you fallen in the past year?”). For older adults who report a fall, clinicians could provide a brief physical activity prescription that specifies frequency (e.g., ≥ 3 times/week), intensity (moderate), and type (e.g., balance, strength, and aerobic exercises), adapted from evidence-based programs. Second, referrals to physical therapists or community-based fall prevention classes should become a standard care pathway after a fall, covered by insurance or public health systems. Third, for older adults fearful of falling, cognitive-behavioral strategies combined with graded activity scheduling can be integrated into routine consultations. Finally, electronic health records could include decision support tools that automatically prompt providers to deliver physical activity advice and follow-up plans when a fall is recorded. These low-cost, scalable interventions can help embed physical activity promotion into everyday clinical practice.

From a policy perspective, integrating physical activity promotion into post-fall care guidelines and expanding access to tailored exercise programs through community initiatives, home-based interventions, and caregiver education may help achieve healthier aging at the population level.

This study has several strengths. We analyzed large, population-based longitudinal datasets with standardized physical activity measures, allowing robust comparisons and improving generalizability. The prospective design reduces the likelihood of reverse causation and supports the temporal relationship between falls, physical activity, and dementia risk.

### Limitations

This study has several limitations. First, physical activity was self-reported, which may have introduced recall bias and misclassification, and we were unable to distinguish between leisure-time and work-related physical activity. However, as most participants were aged ≥ 60 years and likely retired, the influence of work-related physical activity on overall activity levels is expected to be minimal. Second, the definition and recall period for falls differed across cohorts: HRS and ELSA asked participants about falls over the previous two years, whereas SHARE used a six-month recall period. This discrepancy likely led to under-ascertainment of fall history in SHARE and may have introduced heterogeneity in exposure classification, potentially diluting the observed associations when pooling results across cohorts. Third, although we adjusted for a wide range of potential confounders, unmeasured variables such as fall-related hospitalisation and injury severity may still have affected the observed associations. Fourth, although dementia was defined using comparable approaches across cohorts, there were still differences in specific measurements, including self-reported diagnoses, cognitive tests, and functional assessments. These variations may have introduced measurement heterogeneity and affected the comparability of dementia ascertainment across studies. Future studies could benefit from harmonized cognitive assessment initiatives such as the Harmonized Cognitive Assessment Protocol (HCAP) [55], which provides more standardized and detailed measures of cognitive function and dementia across international cohorts. Finally, participants were drawn from high-income countries, limiting the generalisability of our findings to low- and middle-income settings.

Future studies should leverage device-based measures of physical activity, apply biomarker-based definitions of dementia, and include populations from diverse geographic and socioeconomic backgrounds to better elucidate the causal pathways linking falls, physical activity, and dementia risk.

## Conclusion

Falls substantially increase dementia risk among older adults. Higher levels of physical activity, both routinely and after a fall, are associated with lower risks of dementia and future falls, with stronger protective effects at higher activity levels. Promoting physical activity should be prioritized in post-fall care and public health strategies to help preserve cognitive health in aging populations.

## Supplementary Information

Supplementary Material 1.

## Data Availability

The data that support the findings of this study are openly available at: https://hrsdata.isr.umich.edu, https://beta.ukdataservice.ac.uk, https://www.share-eric.eu, accessed on July 2024.

## Abbreviations

HRS: Health and Retirement Study
ELSA: English Longitudinal Study of Ageing
SHARE: Survey of Health, Ageing and Retirement in Europe
PA: Physical activity
BDNF: Brain-derived neurotrophic factor
ADL: Activities of daily living
HR: Hazard ratio
CI: Confidence interval
