# Supplementary material for: Physical activity, falls, and dementia risk in adults aged ≥ 60: evidence from three cohorts
Source: Int J Behav Nutr Phys Act. 2026 May 9;23:69. doi: 10.1186/s12966-026-01926-9 (PMC13330211; doi:10.1186/s12966-026-01926-9)
Supplement: Supplementary file 1 — Supplementary Material 1. [file 12966_2026_1926_MOESM1_ESM.doc]

| Supplementary Table 1. Association of physical activity with risks of incident dementia (pooled) | | | | |
| --- | --- | --- | --- | --- |
| PA | Doctors diagnosed dementia | Combined cognitive and functional impairment | Excl. prior stroke | Excl. dementia ≤2y |
|  | HR (95%CI) | | | |
| **No-fall** |  |  |  |  |
| Inactive | 1.00 (reference) | 1.00 (reference) | 1.00 (reference) | 1.00 (reference) |
| Low | 0.65 (0.55-0.72, p<.001) | 0.62 (0.56-0.70, p<.001) | 0.63 (0.56-0.71, p<.001) | 0.64 (0.57-0.73, p<.001) |
| Moderate | 0.54 (0.45-0.60, p<.001) | 0.53 (0.46-0.59, p<.001) | 0.54 (0.48-0.60, p<.001) | 0.53 (0.47-0.59, p<.001) |
| High | 0.41 (0.34-0.46, p<.001) | 0.42 (0.35-0.48, p<.001) | 0.42 (0.37-0.48, p<.001) | 0.42 (0.37-0.48, p<.001) |
| **Fall** |  |  |  |  |
| Inactive | 1.00 (reference) | 1.00 (reference) | 1.00 (reference) | 1.00 (reference) |
| Low | 0.83 (0.68-1.01, p=.057) | 0.81 (0.67-0.98, p=.034) | 0.75 (0.62-0.91, p=.003) | 0.72 (0.60-0.87, p=.001) |
| Moderate | 0.59 (0.49-0.71, p<.001) | 0.59 (0.49-0.72, p<.001) | 0.58 (0.48-0.70, p<.001) | 0.54 (0.45-0.65, p<.001) |
| High | 0.54 (0.42-0.70, p<.001) | 0.54 (0.42-0.69, p<.001) | 0.52 (0.41-0.67, p<.001) | 0.55 (0.43-0.69, p<.001) |
| Model was adjusted for gender, age, education level, marital status, smoking status, drinking, chronic disease and depressive symptom. | | | | |

| Supplementary Table 2. Association between physical activity and incident dementia (considering competing risk of death) | | |
| --- | --- | --- |
| Physical Activity | HRS | SHARE |
|  | HR (95%CI) | |
| **No-fall** |  |  |
| Inactive | 1.00 (reference) | 1.00 (reference) |
| Low | 0.66 (0.48-0.83, p<.001) | 0.64 (0.55-0.74, p<.001) |
| Moderate | 0.60 (0.49-0.77, p<.001) | 0.57 (0.50-0.65, p<.001) |
| High | 0.58 (0.48-0.74, p<.001) | 0.45 (0.38-0.52, p<.001) |
| **Fall** |  |  |
| Inactive | 1.00 (reference) | 1.00 (reference) |
| Low | 0.91(0.71-1.15, p=.420) | 0.86 (0.60-1.23, p=.410) |
| Moderate | 0.69 (0.54-0.89, p=.004) | 0.55 (0.39-0.77, p<.001) |
| High | 0.64 (0.48-0.86, p=.003) | 0.55 (0.35-0.86, p=.009) |
| HRS, Health and Retirement Study; SHARE, Survey of Health, Ageing and Retirement in Europe. Model was adjusted for gender, age, education level, marital status, smoking status, drinking, chronic disease and depressive symptom. | | |

| Supplementary Table 3. Association between post-fall physical activity and risk of dementia in subgroups（pooled）. | | | | | | | | | | | | | | | |
| --- | --- | --- | --- | --- | --- | --- | --- | --- | --- | --- | --- | --- | --- | --- | --- |
| **Variables** | **Inactive (reference)** | **Low** | **HR (95%CI)** | **P** | **P for int** |  | **Moderate** | **HR (95%CI)** | **P** | **P for int** |  | **High** | **HR (95%CI)** | **P** | **P for int** |
| Age |  |  |  |  | 0.107 |  |  |  |  | 0.001 |  |  |  |  | 0.021 |
| 60-69 | 58/347 | 44/413 | 0.55 (0.37-0.82) | 0.003 |  |  | 35/753 | 0.23 (0.15-0.35) | <.001 |  |  | 17/399 | 0.20 (0.12-0.35) | <.001 |  |
| 70-79 | 141/704 | 79/598 | 0.53 (0.41-0.70) | <.001 |  |  | 105/865 | 0.49 (0.38-0.63) | <.001 |  |  | 51/459 | 0.42 (0.30-0.58) | <.001 |  |
| ≥80 | 166/617 | 90/341 | 0.79 (0.61-1.02) | 0.073 |  |  | 96/446 | 0.55 (0.43-0.71) | <.001 |  |  | 40/211 | 0.49 (0.35-0.70) | <.001 |  |
| Gender |  |  |  |  | 0.003 |  |  |  |  | 0.004 |  |  |  |  | 0.490 |
| Male | 263/1166 | 119/863 | 0.47 (0.37-0.58) | <.001 |  |  | 132/1329 | 0.32 (0.26-0.39) | <.001 |  |  | 62/630 | 0.31 (0.23-0.40) | <.001 |  |
| Female | 102/502 | 94/489 | 0.80 (0.60-1.06) | 0.119 |  |  | 104/735 | 0.54 (0.41-0.70) | <.001 |  |  | 46/439 | 0.37 (0.26-0.52) | <.001 |  |
| Marital status |  |  |  |  | 0.426 |  |  |  |  | 0.115 |  |  |  |  | 0.536 |
| Married or partnered | 149/748 | 112/764 | 0.63 (0.49-0.80) | <.001 |  |  | 109/1195 | 0.35 (0.27-0.44) | <.001 |  |  | 66/676 | 0.36 (0.27-0.49) | <.001 |  |
| Other marital status | 216/920 | 101/588 | 0.54 (0.43-0.69) | <.001 |  |  | 127/869 | 0.46 (0.37-0.58) | <.001 |  |  | 42/393 | 0.31 (0.23-0.44) | <.001 |  |
| Education |  |  |  |  | 0.471 |  |  |  |  | 0.214 |  |  |  |  | 0.456 |
| Below high school | 194/784 | 83/461 | 0.61 (0.47-0.79) | <.001 |  |  | 78/663 | 0.37 (0.29-0.49) | <.001 |  |  | 24/252 | 0.27 (0.17-0.41) | <.001 |  |
| High school | 130/710 | 106/719 | 0.63 (0.48-0.81) | <.001 |  |  | 114/987 | 0.46 (0.36-0.59) | <.001 |  |  | 49/521 | 0.37 (0.27-0.52) | <.001 |  |
| College or above | 41/174 | 24/172 | 0.47 (0.28-0.78) | 0.004 |  |  | 44/414 | 0.31 (0.20-0.48) | <.001 |  |  | 35/296 | 0.36 (0.23-0.57) | <.001 |  |
| Drinking |  |  |  |  | 0.233 |  |  |  |  | 0.771 |  |  |  |  | 0.700 |
| No | 269/1118 | 136/696 | 0.65 (0.53-0.80) | <.001 |  |  | 135/998 | 0.42 (0.35-0.52) | <.001 |  |  | 54/430 | 0.38 (0.28-0.51) | <.001 |  |
| Yes | 96/550 | 77/656 | 0.53 (0.39-0.72) | <.001 |  |  | 101/1066 | 0.40 (0.30-0.53) | <.001 |  |  | 54/639 | 0.34 (0.25-0.48) | <.001 |  |
| Smoking |  |  |  |  | 0.492 |  |  |  |  | 0.540 |  |  |  |  | 0.876 |
| Non-smoker | 168/741 | 92/630 | 0.52 (0.40-0.67) | <.001 |  |  | 127/979 | 0.42 (0.33-0.53) | <.001 |  |  | 53/504 | 0.33 (0.24-0.45) | <.001 |  |
| Former smoker | 162/734 | 101/585 | 0.60 (0.47-0.77) | <.001 |  |  | 93/900 | 0.35 (0.27-0.45) | <.001 |  |  | 49/506 | 0.31 (0.23-0.43) | <.001 |  |
| Current smoker | 35/193 | 20/137 | 0.69 (0.40-1.20) | 0.190 |  |  | 16/185 | 0.38 (0.21-0.69) | 0.002 |  |  | 6/59 | 0.37 (0.15-0.88) | 0.024 |  |
| Hypertension |  |  |  |  | 0.636 |  |  |  |  | 0.162 |  |  |  |  | 0.077 |
| No | 112/512 | 69/463 | 0.54 (0.40-0.73) | <.001 |  |  | 85/842 | 0.33 (0.25-0.44) | <.001 |  |  | 42/498 | 0.26 (0.18-0.37) | <.001 |  |
| Yes | 253/1156 | 144/889 | 0.59 (0.48-0.72) | <.001 |  |  | 151/1222 | 0.43 (0.35-0.52) | <.001 |  |  | 66/571 | 0.39 (0.30-0.51) | <.001 |  |
| Diabetes |  |  |  |  | 0.061 |  |  |  |  | 0.004 |  |  |  |  | 0.029 |
| No | 260/1145 | 154/1031 | 0.52 (0.42-0.63) | <.001 |  |  | 181/1687 | 0.34 (0.28-0.41) | <.001 |  |  | 86/907 | 0.29 (0.23-0.37) | <.001 |  |
| Yes | 105/523 | 59/321 | 0.74 (0.54-1.02) | 0.066 |  |  | 55/377 | 0.60 (0.43-0.83) | 0.002 |  |  | 22/162 | 0.53 (0.33-0.84) | 0.007 |  |
| Cancer |  |  |  |  | 0.189 |  |  |  |  | 0.295 |  |  |  |  | 0.131 |
| No | 319/1378 | 180/1145 | 0.55 (0.46-0.66) | <.001 |  |  | 204/1788 | 0.38 (0.31-0.45) | <.001 |  |  | 90/910 | 0.31 (0.24-0.39) | <.001 |  |
| Yes | 46/290 | 33/207 | 0.74 (0.47-1.17) | 0.196 |  |  | 32/276 | 0.49 (0.31-0.77) | 0.002 |  |  | 18/159 | 0.49 (0.28-0.84) | 0.010 |  |
| Lung disease |  |  |  |  | 0.050 |  |  |  |  | 0.065 |  |  |  |  | 0.963 |
| No | 312/1388 | 179/1184 | 0.53 (0.44-0.64) | <.001 |  |  | 206/1864 | 0.37 (0.31-0.44) | <.001 |  |  | 100/984 | 0.33 (0.26-0.41) | <.001 |  |
| Yes | 53/280 | 34/168 | 0.87 (0.57-1.35) | 0.540 |  |  | 30/200 | 0.59 (0.37-0.93) | 0.022 |  |  | 8/85 | 0.34 (0.16-0.71) | 0.004 |  |
| Heart disease |  |  |  |  | 0.186 |  |  |  |  | 0.107 |  |  |  |  | <.001 |
| No | 216/968 | 139/943 | 0.54 (0.43-0.66) | <.001 |  |  | 159/1488 | 0.36 (0.29-0.44) | <.001 |  |  | 63/782 | 0.26 (0.19-0.34) | <.001 |  |
| Yes | 149/700 | 74/409 | 0.67 (0.51-0.89) | 0.006 |  |  | 77/576 | 0.48 (0.37-0.64) | <.001 |  |  | 45/287 | 0.56 (0.40-0.78) | <.001 |  |
| Stroke |  |  |  |  | 0.728 |  |  |  |  | 0.459 |  |  |  |  | 0.184 |
| No | 279/1380 | 176/1200 | 0.58 (0.48-0.70) | <.001 |  |  | 201/1883 | 0.40 (0.33-0.48) | <.001 |  |  | 91/992 | 0.33 (0.26-0.42) | <.001 |  |
| Yes | 86/288 | 37/152 | 0.64 (0.43-0.94) | 0.024 |  |  | 35/181 | 0.49 (0.33-0.72) | <.001 |  |  | 17/77 | 0.50 (0.30-0.84) | 0.009 |  |
| Arthritis |  |  |  |  | 0.600 |  |  |  |  | 0.556 |  |  |  |  | 0.373 |
| No | 92/447 | 71/457 | 0.63 (0.46-0.86) | 0.003 |  |  | 91/856 | 0.37 (0.28-0.50) | <.001 |  |  | 41/471 | 0.30 (0.21-0.43) | <.001 |  |
| Yes | 273/1221 | 142/895 | 0.55 (0.45-0.68) | <.001 |  |  | 145/1208 | 0.41 (0.33-0.50) | <.001 |  |  | 67/598 | 0.36 (0.27-0.47) | <.001 |  |
| Osteoporosis |  |  |  |  | 0.301 |  |  |  |  | 0.423 |  |  |  |  | 0.748 |
| No | 239/1154 | 162/1043 | 0.60 (0.49-0.74) | <.001 |  |  | 182/1603 | 0.40 (0.33-0.49) | <.001 |  |  | 84/884 | 0.33 (0.26-0.42) | <.001 |  |
| Yes | 126/514 | 51/309 | 0.50 (0.36-0.70) | <.001 |  |  | 54/461 | 0.36 (0.26-0.50) | <.001 |  |  | 24/185 | 0.36 (0.24-0.57) | <.001 |  |
| Depressive symptom |  |  |  |  | 0.785 |  |  |  |  | 0.094 |  |  |  |  | 0.134 |
| No | 167/870 | 127/898 | 0.58 (0.46-0.73) | <.001 |  |  | 145/1467 | 0.36 (0.29-0.46) | <.001 |  |  | 75/858 | 0.32 (0.24-0.42) | <.001 |  |
| Yes | 198/798 | 86/454 | 0.61 (0.47-0.78) | <.001 |  |  | 91/597 | 0.50 (0.39-0.64) | <.001 |  |  | 33/211 | 0.46 (0.31-0.66) | <.001 |  |
